# Comparative effectiveness of biguanides versus SGLT2 inhibitors on cardiovascular and cerebrovascular events, diabetic nephropathy, retinopathy, neuropathy, and treatment expenditures in patients with type 2 diabetes

**DOI:** 10.1371/journal.pone.0336038

**Published:** 2025-11-06

**Authors:** Eiji Nakatani, Hiromitsu Ohno, Takayoshi Nagahama, Toru Tonoike, Hiromichi Yui, Tatsunori Satoh, Taku Matsunaga, Daito Funaki, Chikara Ueki, Emi Ohata, Akinori Miyakoshi, Ataru Igarashi, Yoshihiro Tanaka, Hideaki Kaneda, Hiraku Kumamaru, Akira Sugawara

**Affiliations:** 1 Graduate School of Public Health, Shizuoka Graduate University of Public Health, Shizuoka, Japan; 2 Allied Medical K.K., Tokyo, Japan; 3 Department of Biostatistics and Data Science, Graduate School of Medical Science Nagoya City University, Nagoya, Japan; 4 Institute of Humanistic Social Medicine, Tokyo, Japan; 5 Shizuoka General Hospital, Shizuoka, Japan,; 6 Department of Academic Services, 4DIN Ltd., Tokyo, Japan; 7 Graduate School of Pharmaceutical Sciences, University of Tokyo, Tokyo, Japan; 8 Okinaka Memorial Institute for Medical Research, Tokyo, Japan; 9 Department of Health Data Science, Graduate School of Data Science Yokohama City University, Yokohama, Japan; University of Udine, ITALY

## Abstract

**Background:**

Sodium–glucose cotransporter-2 (SGLT2) inhibitors are increasingly recommended as first-line treatment for type 2 diabetes mellitus (T2DM), but head-to-head data comparing them with metformin, the canonical biguanide, remain sparse in Japan.

**Objective:**

To compare the long-term effectiveness and cost of initiating treatment with a biguanide versus an SGLT2 inhibitor, excluding the alternative class for 12 months but permitting other antidiabetic drugs, on a composite of major cardio-cerebrovascular events and all-cause death, and a composite of diabetic complications.

**Methods:**

We emulated a new-user cohort trial using the Shizuoka Kokuho Database (2014–2021). Patients initiating treatment with either a biguanide or an SGLT2 inhibitor, while avoiding the alternative class during the first 12 months but allowing other glucose-lowering agents, were included. Follow-up began at treatment initiation; those who received the comparator drug within 12 months were excluded. After 1:1 propensity-score matching on demographic, clinical, laboratory, and lifestyle variables, cause-specific Cox models estimated hazard ratios (HRs). Daily medication costs were compared.

**Results:**

After matching, 1,246 patients (623 per group) were followed for a median of 2.9 years (maximum 7.2 years). Cardio-cerebrovascular composite: 44/623 biguanide users (7.1%) and 35/623 SGLT2 inhibitor users (5.6%) experienced a first event (HR 0.80, 95% CI 0.51–1.24). Diabetic complications: 86/623 (13.8%) vs. 78/623 (12.5%) (HR 0.88, 95% CI 0.70–1.13). Median daily drug cost was 124.7 JPY for biguanides and 184.0 JPY for SGLT2 inhibitors (P < 0.001).

**Conclusion:**

Using a large-scale regional database from Japan, we found that among adults with type 2 diabetes without prior major cardiac or renal disease, first-line treatment with an SGLT2 inhibitor did not reduce risks of cardio-cerebrovascular events, mortality, or complications compared with metformin, and cost about 50% more.

## Introduction

Globally, type 2 diabetes mellitus (T2DM) afflicts an estimated 589 million adults (aged 20–79 years)and is projected to climb to exceed 853 million by 2050, with cardiovascular and cerebrovascular events accounting for the majority of diabetes-related deaths and expenditure [1]. These macro-vascular sequelae underscore the clinical and economic stakes of a patient’s very first glucose-lowering prescription.

For more than two decades, international guidance—exemplified by the 2025 American Diabetes Association “Pharmacologic Approaches to Glycemic Treatment” consensus—has positioned metformin, a biguanide, as foundational therapy because of its durable glycaemic control, tolerable safety profile and negligible cost [2]. Conversely, large cardiovascular-outcome trials such as EMPA-REG OUTCOME demonstrated that sodium–glucose cotransporter-2 (SGLT2) inhibitors confer significant reductions in cardiovascular death, heart-failure hospitalisation and renal decline, benefits that transcend glucose lowering and have prompted many societies to promote these agents early in the treatment algorithm [3,4]. Recent real-world analyses are mixed: a U.S. cohort study of > 25 000 initiators reported comparable risks for myocardial infarction, stroke and mortality but a lower hazard for heart-failure events with first-line SGLT2 inhibition versus metformin [5,6]. A meta-analysis published in 2024 likewise found no material difference in major adverse cardiovascular events between the two classes, although SGLT2 inhibitors retained their cardiorenal advantages [7].

Evidence from Japan is even sparser. Our recent nationwide study showed that biguanides and dipeptidyl-peptidase-4 inhibitors produced similar cardio-cerebrovascular outcomes but markedly different drug costs [8]. Another Japanese database analysis confirmed that selecting costlier agents as first-line therapy almost doubled annual medical expenditure without lowering complication rates [9]. Yet no head-to-head evaluation has compared therapies with biguanide and SGLT2 inhibitor in contemporary Japanese practice.

This study employed a new‑user cohort design to determine whether, in Japanese adults with type 2 diabetes without prior major cardiac or renal disease, initiating treatment with a biguanide versus an SGLT2 inhibitor—while refraining from using the alternative class during the first year yet allowing any other concomitant medications—produces long‑term differences in the hazards of major cardio‑cerebrovascular events, diabetic complications, or total pharmacotherapy costs.

## Materials and methods

### Data source, study design, and study population

We conducted a retrospective cohort study employing a new-user design [10] with data extracted from the Shizuoka Kokuho Database (SKDB), version 2023 [11], the observation window spanned from 1 April 2012–30 September 2021. The SKDB houses comprehensive medical and long-term-care insurance records for more than 2.3 million residents of Shizuoka Prefecture, providing a demographically diverse sample [11]. Its utility for evaluating drug effectiveness and safety has been demonstrated in prior investigations [8,12,13]. Database access for the present study was granted from January 18 and 27, 2024.

Eligible individuals were beneficiaries of either National Health Insurance (< 75 years) or the Latter-Stage Elderly Medical Care System (≥ 75 years) who had a documented diagnosis of T2DM. Inclusion required receipt of a first-line antidiabetic prescription following a 1-year baseline assessment period and completion of a health check-up within the 6 months preceding treatment initiation. We excluded patients with any of the following during baseline; relevant genetic disorders; prior cerebrovascular or cardiac events; cancer; dialysis; use of glucagon or insulin therapy; or participation in home self-injection training.

### First-line antidiabetic therapies

**Fig 1:**
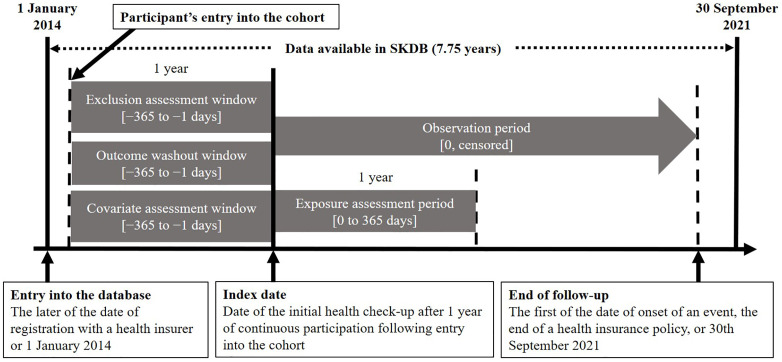
Study schema. SGLT2 = sodium–glucose cotransporter-2; SKDB = Shizuoka Kokuho Database. Cohort entry was defined as the later of either the beneficiary’s enrollment date with the health insurer or 1 January 2014. The index date corresponded to the day on which participants were first prescribed either an SGLT2 inhibitor or a biguanide. Follow-up extended from the index date to the earliest of: the study’s end (30 September 2021), withdrawal from the health-insurance system, or the occurrence of a study outcome.

We assessed two mutually exclusive first-line monotherapies: a biguanide (metformin hydrochloride or buformin hydrochloride) and a SGLT2 inhibitor. The specific National Health Insurance drug codes used to identify eligible prescriptions within the SKDB are provided in S1 Table. For each participant, the index date was defined as the day the initial antidiabetic agent was dispensed (Fig 1). Individuals were then assigned to one of two analytic cohorts according to this index prescription, which served as the primary exposure variable. Patients were excluded if they failed to attend any clinic visit for antidiabetic management for more than six consecutive months during the first year after the index date.

### Potential confounders and additional covariates

To minimise residual confounding when comparing first-line SGLT2 inhibitor and biguanide therapy, we adjusted for an extensive set of demographic, clinical, and lifestyle factors. Demographic variables included sex and age. Baseline comorbidities comprised hypertension, dementia, renal disease, rheumatic disease, liver disease, and chronic pulmonary disease, based on the Charlson and Elixhauser comorbidity indices [14,15]. Detailed ICD-10 code definitions are provided in S2 Table. Concomitant medications taken into account were antihypertensive and lipid-lowering agents.

Lifestyle and anthropometric factors were: body-mass index; glycated haemoglobin (HbA1c); habitual physical activity (walking or other exercise ≥ 1 h per week); weight gain > 10 kg since age 20 years; current smoking; and heavy alcohol use. Heavy drinking was defined as daily consumption of alcoholic equivalent to more than 360 mL of Japanese rice wine (sake, ≈ 43 g of pure alcohol). A current smoker was defined as an individual who had smoked ≥ 100 cigarettes or for ≥ 6 months and had smoked within the past month. These variables or their derivatives are routinely collected and defined according to standardized criteria used in the nationwide health check-up system in Japan [16].

Laboratory and physiological measurements adjusted for included γ-glutamyl transferase, systolic and diastolic blood pressure, estimated glomerular filtration rate, aspartate aminotransferase (AST), alanine aminotransferase (ALT), low- and high-density lipoprotein (LDL and HDL) cholesterol, triglycerides, and uric acid. The Anatomical Therapeutic Chemical codes used to identify other antidiabetic agents for cohort construction are detailed in S3 Table.

### Outcomes

The primary outcome was the time from the index date to the first event in a composite endpoint consisting of any cerebrovascular event, cardiac event, or all-cause mortality, identified with the diagnosis and procedure codes listed in S4 Table. Secondary outcomes were the times from the index date to the initial occurrence of any diabetes-related complication, including diabetic nephropathy, renal failure, diabetic retinopathy, and diabetic peripheral neuropathy. These events were defined strictly using pre-specified diagnosis codes, as listed in S5 Table. Participants who, within six months of the index date, were hospitalised or experienced a cerebrovascular event, a cardiac event, cancer, initiation of dialysis, or death were censored at that time, providing a six-month grace period before comparing first-line SGLT2 inhibitor and biguanide treatments.

Cardiac events were defined as hospital admissions coded with International Classification of Diseases, 10th Revision (ICD-10): I20, I21, I22, I24, I25, or I50—covering acute and chronic coronary syndromes and heart failure—and included major revascularisation procedures such as percutaneous coronary intervention or coronary artery bypass grafting. This definition follows standard clinical-epidemiological practice in diabetes research.

Stroke was identified with ICD-10 I60, I61, I62, I63, or I64, while other cerebrovascular events were captured with Japanese health-care procedure codes 8838736, 8838748, and 8838750. This combined use of international and Japan-specific codes ensured comprehensive, context-appropriate ascertainment of cerebrovascular outcomes [17,18].

### Medication cost analysis

To quantify medication expenditures in the SGLT2 inhibitor and biguanide cohorts, we computed each patient’s average daily outlay in Japanese yen (JPY) across the entire observation period. Expressing cost on a per-day basis permits an equitable comparison of treatment expenses within fixed time frames, irrespective of follow-up duration. Focusing on the mean daily charge rather than the cumulative sum minimises bias from unequal observation lengths and provides a precise appraisal of the financial burden associated with each antihyperglycaemic regimen.

### Statistical analysis

Continuous variables are presented as means ± standard deviations or median (inter-quartile range [IQR]), and categorical variables as counts with percentages. Propensity scores were generated for each participant via multivariable logistic regression that incorporated all prespecified confounders; covariates for which reliable effect-size estimation was infeasible were omitted. Study cohorts were constructed with one-to-*k* nearest-neighbour matching on the logit of the propensity score, applying a caliper of 0.20. The integer *k* was chosen (≤ 10) so that at least 90% of each treatment group was retained after matching. Group balance was evaluated using absolute standardised mean differences (ASMDs), accepting an ASMD < 0.1 as adequate balance.

Time-to-event outcomes were examined with cumulative-incidence curves. Between-group differences were tested with the log-rank test for overall survival and the composite endpoint, and with Gray’s test [19] for other outcomes while treating death as a competing risk. Cumulative-incidence point estimates with 95% confidence intervals (CIs) were obtained, and hazard ratios (HRs) with 95% CIs were estimated using cause-specific Cox regression.

Daily medication cost was compared between the SGLT2 inhibitor and biguanide groups using the Wilcoxon rank-sum test; the mean difference and its 95% CI were derived from a univariable regression model. Sensitivity analyses repeated Gray’s test and cause-specific Cox modelling among participants with clinic attendance of at least 9 months, and again among those with a full 12-month follow-up. Prespecified subgroup analyses were conducted for sex (male, female), age (< 70 y, 70–79.9 y, ≥ 80 y), body-mass index (< 25 kg m ⁻ ², ≥ 25 kg m ⁻ ²), hypertension (yes, no), liver disease (yes, no), renal disease (yes, no), and use of lipid-lowering agents (yes, no). Missing values were left unimputed. Two-sided P-values < 0.05 were considered statistically significant. All statistical analyses were performed with EZR version 1.61 (Saitama Medical Center, Jichi Medical University, Tochigi, Japan) [20]. SAS version 9.4 (SAS Institute Inc., Cary, NC, USA) was used only for dataset preparation (e.g., data cleaning and variable construction).

### Ethical considerations

This investigation—an observational, retrospective comparison of first-line SGLT2 inhibitor and biguanide use—relied solely on SKDB records that had been irreversibly anonymised before analysis, thereby safeguarding participant privacy and confidentiality [11]. The study protocol (SGUPH_2021_001_078) received approval from the Ethics Committee of Shizuoka Graduate University of Public Health, confirming that all procedures conformed to relevant ethical standards, national regulations, and the principles of the Declaration of Helsinki. Owing to the study’s retrospective design and strict adherence to Japanese medical-ethics guidelines, the requirement for individual informed consent was formally waived.

## Results

### Characteristics of the participants

**Fig 2:**
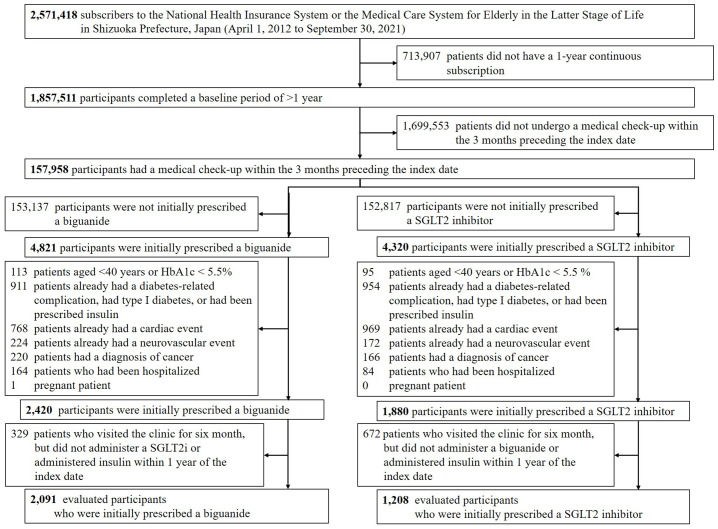
Flow diagram of participant selection.

Fig 2 presents the study flow diagram. Baseline characteristics before and after propensity-score matching are summarised in S6 Table and Table 1, respectively. Prior to matching, the biguanide and SGLT2 inhibitor cohorts differed with respect to age at treatment initiation, body-mass index, AST, ALT and LDL cholesterol measured at health check-up (standardised mean difference > 0.15; S6 Table). Propensity matching was performed on the distribution of covariates in the biguanide cohort, after which all baseline variables were well balanced between the two treatment groups (standardised mean difference < 0.10; Table 1), indicating successful comparability.

**Table 1:** Baseline characteristics of the matched cohort.

Variable	Category (unit)	After matching		ASMD
		Biguanide	SGLT2 inhibitor	
		N = 623	N = 623	
Sex	Male	336 (53.8)	335 (53.6)	0.003
Age	(years)	67.56 (8.10)	67.61 (7.97)	0.007
	40 to 49.9 years	26 (4.2)	26 (4.2)	<0.001
	50 to 59.9 years	58 (9.3)	58 (9.3)	
	60 to 69.9 years	268 (42.9)	268 (42.9)	
	70 to 79.9 years	235 (37.6)	235 (37.6)	
	≥80 years	38 (6.1)	38 (6.1)	
**Comorbidities**				
Hypertension	Presence	387 (61.9)	376 (60.2)	0.036
Dementia	Presence	4 (0.6)	4 (0.6)	<0.001
Renal disease	Presence	6 (1.0)	6 (1.0)	<0.001
Rheumatic disease	Presence	11 (1.8)	10 (1.6)	0.012
Liver disease	Presence	140 (22.4)	154 (24.6)	0.053
Chronic pulmonary disease	Presence	113 (18.1)	112 (17.9)	0.004
**Medication**				
Anti-hypertensive agent	Yes	348 (55.7)	333 (53.3)	0.048
Lipid-lowering agent	Yes	332 (53.1)	331 (53.0)	0.003
**Medical checkup**				
BMI	(kg/m^2^)	25.52 (4.03)	25.70 (4.06)	0.043
	<18.50 kg/m^2^	16 (2.6)	11 (1.8)	0.074
	18.50 to 21.99 kg/m^2^	91 (14.6)	84 (13.4)	
	22.00 to 24.99 kg/m^2^	192 (30.7)	197 (31.5)	
	25.00 to 29.99 kg/m^2^	253 (40.5)	252 (40.3)	
	≥30.00 kg/m^2^	73 (11.7)	81 (13.0)	
HbA1c	(%)	7.53 (1.33)	7.56 (1.26)	0.025
	<6.00%	14 (2.2)	12 (1.9)	0.062
	6.00 to 6.49%	62 (9.9)	63 (10.1)	
	6.50 to 6.99%	147 (23.5)	138 (22.1)	
	7.00 to 7.99%	254 (40.6)	249 (39.8)	
	≥8.00%	148 (23.7)	163 (26.1)	
Walking or physical exercise for >1 hour/week	Yes	279 (44.6)	275 (44.0)	0.013
Current smoker	Yes	101 (16.2)	98 (15.7)	0.013
Heavy alcohol drinking	Yes	38 (6.1)	42 (6.7)	0.026
GGT	(U/L)	52.45 (73.06)	52.22 (66.31)	0.003
Systolic blood pressure	(mmHg)	134.28 (16.88)	133.85 (15.83)	0.026
Diastolic blood pressure	(mmHg)	76.25 (11.35)	76.62 (10.78)	0.033
Estimated GFR	(mL/min/1.73 m^2^)	73.47 (15.88)	72.64 (16.07)	0.052
AST	(U/L)	28.84 (15.73)	29.61 (18.35)	0.045
ALT	(U/L)	31.90 (21.98)	32.76 (22.50)	0.039
LDL-cholesterol	(mg/dL)	122.44 (31.48)	122.96 (30.27)	0.017
HDL-cholesterol	(mg/dL)	55.55 (13.75)	55.59 (15.04)	0.003
Triglycerides	(mg/dL)	156.93 (108.29)	161.63 (109.96)	0.043
Uric acid	(mg/dL)	5.26 (1.26)	5.26 (1.28)	0.001

ALT, alanine aminotransferase; AST, aspartate aminotransferase; BMI, body mass index; SGLT2, sodium–glucose cotransporter 2; GFR, glomerular filtration rate; GGT, gamma-glutamyl transpeptidase; HbA1c, hemoglobin A1c; LDL, low-density lipoprotein; HDL, high-density lipoprotein; ASMD: absolute standardized mean difference.

### Prescriptions of antidiabetic medication and clinic visits following propensity score matching

The antidiabetic agents dispensed before propensity matching and the corresponding clinic-visit counts are summarised in S7 Table. Table 2 details prescriptions issued in the first year after the index date after propensity-score matching for individuals who initiated therapy with either a biguanide (n = 623) or an SGLT2 inhibitor (n = 623).

**Table 2:** Antidiabetic prescriptions and healthcare visits during the first year after the index date in the matched cohorts.

Variable	After matching	
	Biguanide (n = 623)	SGLT2 inhibitor (n = 623)
Biguanides	623 (100.0)	0
SGLT2 inhibitors	0	623 (100.0)
Insulin	0	0
GLP-1 receptor agonists	1 (0.2)	5 (0.8)
DPP-4 inhibitors	389 (62.4)	372 (59.7)
Alpha-glucosidase inhibitors	41 (6.6)	54 (8.7)
Thiazolidinediones (also known as glitazones)	28 (4.5)	55 (8.8)
Rapid-acting secretagogues (meglitinides, also known as glinides)	26 (4.2)	37 (5.9)
Sulfonylureas	78 (12.5)	136 (21.8)
Number of healthcare visits (month, per year)	10.6 ± 1.9	10.7 ± 1.8

DPP-4, dipeptidyl peptidase 4; GLP, glucagon-like peptide-1; SGLT2, sodium–glucose cotransporter 2.

After matching, all participants (100%) remained on their original index drug, and none required insulin. Concomitant use of other glucose-lowering agents was uncommon overall. GLP-1 receptor agonists were prescribed for only 0.2% of patients in the biguanide cohort and 0.8% of those in the SGLT2 inhibitor cohort. DPP-4 inhibitors were co-prescribed in roughly three-fifths of each group (62.4% versus 59.7%). Small differences were observed in the prevalences of α-glucosidase inhibitors (6.6% versus 8.7%) and thiazolidinediones (4.5% versus 8.8%). Rapid-acting secretagogues (meglitinides) were used with comparable frequency (4.2% versus 5.9%), whereas sulfonylureas were somewhat more common among SGLT2 inhibitor recipients (21.8% versus 12.5%).

Healthcare utilisation was similar between groups: the biguanide cohort recorded a mean (± SD) of 10.6 ± 1.9 visits per year, and the SGLT2 inhibitor cohort 10.7 ± 1.8 visits, indicating equivalent post-index engagement with clinical services.

### Cardiac and cerebrovascular outcomes

The propensity-matched cohort comprised 623 recipients of biguanide monotherapy and 623 recipients of an SGLT2 inhibitor, yielding a 1:1 matching ratio and a median follow-up of 2.9 years (maximum 7.2 years). Cardiac events occurred in 21 participants (3.4%) in the biguanide group and 18 (2.9%) in the SGLT2 inhibitor group, whereas cerebrovascular events were observed in 12 (1.9%) and 10 (1.6%) participants, respectively. All-cause mortality was recorded for 17 individuals (2.7%) treated with biguanides and 11 (1.8%) treated with SGLT2 inhibitors. Consequently, the composite endpoint of “first cardiac event, first cerebrovascular event, or death” was reached by 44 participants (7.1%) in the biguanide cohort and 35 (5.6%) in the SGLT2 inhibitor cohort (Table 3).

**Table 3:** Comparison of the outcomes of participants who were prescribed biguanide or a SGLT2 inhibitor.

Outcome	Exposure	Events, number (%)	Cumulative incidence after 3 years		*P*-value
			Rate (%)		95% confidence interval
Composite event^**†**^	Biguanide (n = 623)	44 (7.1)	5.9	4.1 - 8.5	0.314
	SGLT2 inhibitor (n = 623)	35 (5.6)	5.5	3.7–8.2	
Cardiac event^*^	Biguanide (n = 623)	21 (3.4)	3.1	1.8 - 5.0	0.638
	SGLT2 inhibitor (n = 623)	18 (2.9)	2.6	1.4 - 4.4	
Cerebrovascular event^*^	Biguanide (n = 623)	12 (1.9)	1.4	0.6 - 2.8	0.677
	SGLT2 inhibitor (n = 623)	10 (1.6)	1.9	0.9 - 3.6	
Death^**†**^	Biguanide (n = 623)	17 (2.7)	2.1	1.1 - 4.0	0.280
	SGLT2 inhibitor (n = 623)	11 (1.8)	1.2	0.5 - 3.0	
Diabetic complication^*^	Biguanide (n = 623)	86 (13.8)	16.8	13.3 - 20.6	0.343
	SGLT2 inhibitor (n = 623)	78 (12.5)	12.9	9.9–16.3	
Diabetic retinopathy^*^	Biguanide (n = 623)	66 (10.6)	13.2	10.1 - 16.7	0.858
	SGLT2 inhibitor (n = 623)	66 (10.6)	10.6	7.9 - 13.8	
Diabetic nephropathy^*^	Biguanide (n = 623)	27 (4.3)	3.9	2.4 - 6.0	0.054
	SGLT2 inhibitor (n = 623)	15 (2.4)	2.4	1.3 - 4.2	
Diabetic neuropathy^*^	Biguanide (n = 623)	6 (1.0)	0.8	0.3-2.0	0.946
	SGLT2 inhibitor (n = 623)	6 (1.0)	0.4	0.1-1.3	
Other conditions^*^	Biguanide (n = 623)	10 (1.6)	1.9	0.9-3.5	0.636
	SGLT2 inhibitor (n = 623)	8 (1.3)	1.0	0.4-2.2	

* Gray’s test was performed. † The log-rank test was performed. SGLT2: sodium–glucose cotransporter 2.

**Fig 3:**
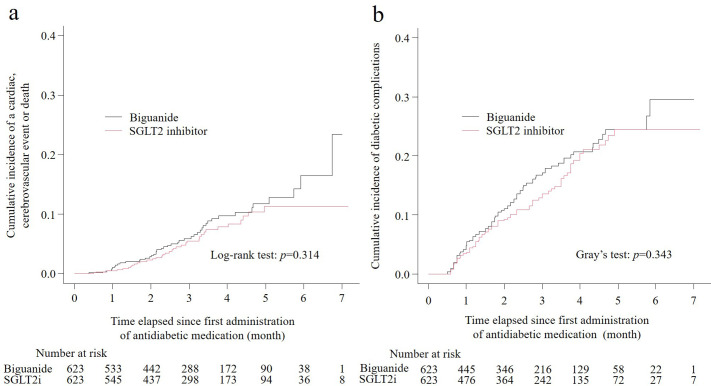
Cumulative incidences of cardiac and cerebrovascular events or mortality, and diabetic complications in the matched cohort. SGLT2: sodium–glucose cotransporter 2. Cumulative incidences of (a) the composite outcome of cardiac or cerebrovascular outcomes or mortality and (b) diabetic complications, including diabetic retinopathy, nephropathy, neuropathy, and other conditions, in the propensity score-matched cohort.

Cumulative-incidence curves (Fig 3a) and the log-rank test showed no statistically significant difference in the composite outcome between treatment groups (P = 0.314). The cause-specific Cox model produced a hazard ratio of 0.80 (95% CI, 0.51–1.24) for biguanide versus SGLT2 inhibitor, indicating comparable risk.

**Fig 4:**
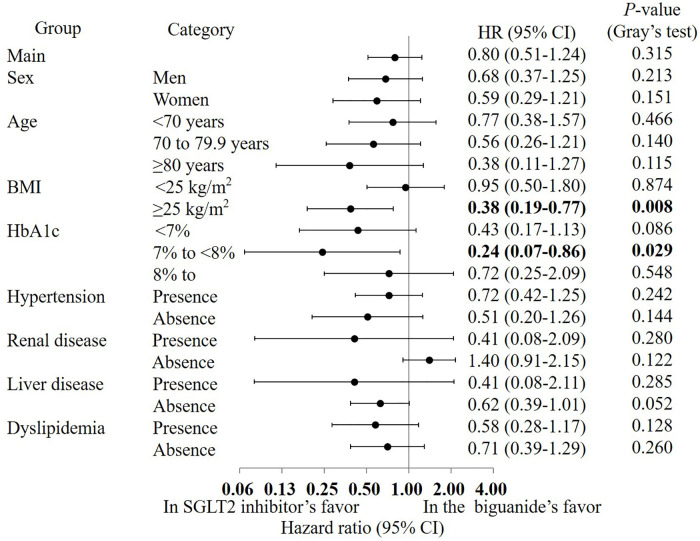
Results of the subgroup analysis of the composite outcome. HR: hazard ratio, CI: confidence interval.

Sensitivity analyses restricted to participants with ≥ 9 months and ≥ 12 months of clinical follow-up likewise demonstrated no between-group differences in the composite endpoint (*P* = 0.115 and *P* = 0.148, respectively; S8 and S9 Tables). The treatment effect was also broadly consistent across the prespecified subgroups depicted in Fig 4. No meaningful heterogeneity was detected by sex, body-mass index, hypertension status, or use of lipid-lowering agents, and liver disease did not materially modify risk overall. Although the point estimates suggested a lower hazard among patients with body-mass index ≥ 25 kg/m^2^ (HR 0.38, 95% CI 0.19–0.77; P = 0.008), and among those with HbA1c of 7% to <8% (HR 0.24, 95% CI 0.07–0.86; P = 0.029).

### Diabetic complications

During follow-up, diabetic complications were documented in 86 participants (13.8%) in the biguanide group and 78 participants (12.5%) in the SGLT2 inhibitor group (P = 0.343; Table 3, Fig 3b). No significant differences were observed in the cumulative incidences of diabetic retinopathy (P = 0.858), nephropathy (P = 0.054), neuropathy (P = 0.946), or other diabetes-related conditions (P = 0.636).

Cause-specific Cox regression produced a HR of 0.88 (95% CI, 0.70–1.13) for any diabetic complication when comparing biguanide with SGLT2 inhibitor use. Corresponding HRs for individual complications were: retinopathy, 0.93 (0.71–1.22); nephropathy, 0.97 (0.61–1.54); neuropathy, 0.76 (0.34–1.67); and other conditions, 1.04 (0.59–1.84).

**Fig 5:**
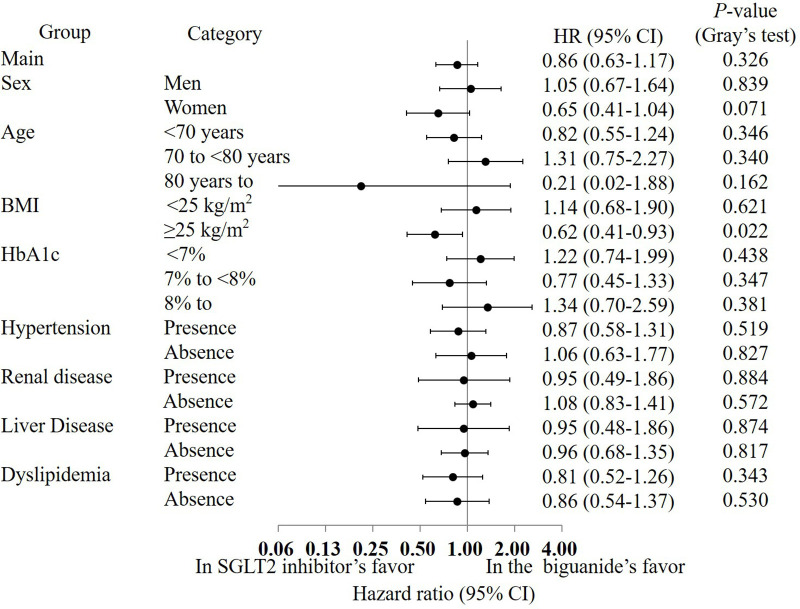
Results of the subgroup analysis of diabetic complications. HR: hazard ratio, CI: confidence interval.

Sensitivity analyses restricted to participants with ≥ 9 months and ≥ 12 months of follow-up likewise showed no between-group differences in the composite complication endpoint (P = 0.405 and P = 0.753, respectively; S8 and S9 Tables). Results were likewise uniform across the prespecified subgroups defined by sex, age, body-mass index, hypertension, liver disease, and concomitant lipid-lowering therapy, with Fig 5 illustrating the absence of any meaningful effect modification.

### Daily cost of antidiabetic medication

The median daily cost of biguanide treatment was 124.7 JPY (IQR 24.6–157.6), whereas SGLT2 inhibitor therapy cost 184.0 JPY (IQR 135.4–220.8). The mean daily expenditure was 110.4 JPY (SD: 80.6) for biguanides and 176.3 JPY (SD: 89.4) for SGLT2 inhibitors, yielding a mean difference of 65.9 JPY (95% CI: 56.5–75.4). A Wilcoxon rank-sum test showed this difference to be statistically significant (p < 0.001), indicating that biguanide therapy remained consistently less expensive—largely reflecting the higher unit price of SGLT2 inhibitors throughout the study period.

## Discussion

Our propensity-matched analysis of more than 1,200 Japanese adults with newly treated T2DM showed that starting an SGLT2 inhibitor conferred no incremental protection against major cardiac or cerebrovascular events, all-cause death, or diabetic complications when compared with first-line biguanide therapy over four years, whereas daily drug cost was almost 50% higher for the SGLT2 regimen. These findings question the clinical and economic value of routine SGLT2 inhibitor use as the very first glucose-lowering agent in an unselected Japanese population.

Metformin remains the clinically and economically preferred first-line therapy owing to its durable glycaemic control, weight neutrality, negligible cost, and demonstrated survival benefit [2]. Although landmark trials such as EMPA-REG OUTCOME prompted early adoption of SGLT2 inhibitors by showing cardiovascular and heart-failure advantages in high-risk populations [3,5], subsequent real-world studies—including our present Japanese cohort—largely report macro-vascular equivalence to metformin, with at best an inconsistent heart-failure signal [21,22]. These findings parallel prior Japanese evidence comparing metformin with DPP-4 inhibitors [8] and underscore that higher-priced alternatives do not necessarily translate into superior first-line cardiovascular protection.

As depicted in Fig 4, treatment with SGLT2 inhibitors reduced the hazard of the primary composite cardio-renal outcome across almost all prespecified strata, whereas in participants without baseline renal disease the hazard ratio crossed unity and numerically favoured biguanide therapy [23,24]. This exception aligns with evidence from large trials and meta-analyses showing that the cardio-renal benefit of SGLT2 inhibition intensifies as estimated glomerular filtration rate declines and albuminuria is present, while the incremental effect diminishes when kidney function is preserved [25–27]. Real-world cohort studies further demonstrate that SGLT2 inhibitors are preferentially initiated in patients with chronic kidney disease or other high-risk profiles, a prescription-channeling pattern that may leave residual confounding even after propensity-score matching with a detailed diabetic retinopathy score [23,28,29]. Consequently, both biologically plausible effect-modification and potential residual bias could explain the hazard-ratio reversal in the renal-disease-absent subgroup, underscoring the need for formal interaction testing and additional sensitivity analyses to confirm the robustness of the Fig 4 finding.

Although statistical significance was not reached, the SGLT2 inhibitor group demonstrated a numerically lower incidence of diabetic nephropathy compared with the biguanide group. This observation is consistent with accumulating evidence that SGLT2 inhibitors exert robust renoprotective effects through mechanisms beyond glycemic control. In the landmark CREDENCE trial, canagliflozin significantly reduced the risk of renal outcomes among patients with type 2 diabetes and established nephropathy [30]. Subsequently, the DAPA-CKD trial demonstrated that dapagliflozin slowed the progression of chronic kidney disease and reduced mortality, even in patients without diabetes [31]. Moreover, a comprehensive meta-analysis has shown that these renoprotective effects are consistent across diverse patient populations and extend beyond glycemic lowering [32]. Taken together, these data support the biological plausibility of the trend observed in our study, which may be mediated by reductions in intraglomerular pressure, improvements in systemic blood pressure and body weight, and attenuation of oxidative stress and inflammation.

Subgroup analysis revealed a significant hazard reduction for the composite outcome among participants with BMI ≥ 25 kg/m² (HR 0.38, 95% CI 0.19–0.77), suggesting a biologically plausible effect modification. Obesity is a well-established driver of insulin resistance and cardiorenal risk, and SGLT2 inhibitors exert multiple favorable metabolic effects—including weight loss, blood pressure reduction, and natriuresis—that may be especially beneficial in this population. These results are consistent with previous studies reporting greater cardiorenal benefit of SGLT2 inhibitors in patients with higher BMI or metabolic risk [33,34]. While our study was not powered to test for formal interaction effects, this finding highlights the potential utility of BMI as a marker for patient selection in personalized diabetes care.

The median daily cost of SGLT2 inhibitors greatly exceeded that of biguanides, echoing earlier Japanese [35] and international cost-modelling studies [4]. Because long-term drug expenditure dominates direct medical costs in T2DM, selection of metformin whenever clinically appropriate remains the fiscally prudent strategy, reserving SGLT2 inhibitors for patients with established cardiorenal indications where their incremental benefit is proven. [4] Large population-based studies demonstrate that generic agents are clinically non-inferior—and in some cardiometabolic settings even superior—to branded products, undermining any therapeutic rationale for premium pricing [36,37]. Nonetheless, prescribing remains skewed toward costlier brands, and evidence shows that value-aligned incentives such as physician bonuses for generics or wider brand–generic copay differentials markedly increase generic uptake and lower spending [38–40].

In our study, the average daily prescription cost of SGLT2 inhibitors was approximately 66 yen higher than that of biguanides. Although this difference may appear modest at the individual level, it may translate into substantial healthcare expenditure when multiplied across the large number of patients receiving long-term antidiabetic therapy. Prior cost-effectiveness analyses in Japan, such as that by Igarashi et al. (2022), have generally concluded that SGLT2 inhibitors are not cost-effective as first-line agents compared with biguanides, under current pricing and effectiveness assumptions [35]. Future research should incorporate real-world data on health outcomes, utilities, and societal costs to comprehensively evaluate the economic value of glucose-lowering therapies in diverse patient populations.

We also observed higher use of thiazolidinediones and sulfonylureas among SGLT2 inhibitor users during the first year after treatment initiation (Table 2), which may reflect channeling bias—a form of allocation bias wherein newer agents are preferentially prescribed to patients with greater perceived treatment needs. A recent meta-analysis demonstrated that GLP-1 receptor agonists reduce atherosclerotic cardiovascular events [41–43], while thiazolidinediones are associated with stroke reduction but increased heart failure risk [44,45], and sulfonylureas are linked to increased cardiovascular risk and mortality [46,47]. Although GLP-1 RAs were infrequently used in our cohort, the more common use of thiazolidinediones and sulfonylureas among SGLT2 inhibitor users may have attenuated or confounded treatment effects. While our propensity-score model adjusted for baseline covariates, these findings underscore the need for future studies using time-varying exposure or marginal structural models to isolate drug-specific effects in real-world settings.

In the primary analysis we censored nephropathy arising within 6 months of the index date, leaving 42 adjudicated events (metformin = 27, SGLT2i = 15) and an HR of 0.97 (95% CI 0.61–1.54). With a two-sided α = 0.05, the Schoenfeld approximation [48] yields a mere 4% power for this effect size—and only 30% even for a clinically relevant 30% reduction (HR 0.70). Reaching the canonical 80% power would demand 62 events, which, at the 4-year cumulative incidence of 4.3% in the control arm, translates to roughly 780 participants per group, assuming no attrition. The sensitivity analysis, which instead censored nephropathy diagnosed within 12 months, observed 25 events (17 vs 8) (P = 0.034; S8 Table) and produced an HR of 0.56. Under the same α and effect-size assumptions, 80% power would require 94 events, equating to about 2,100 per group at the observed average incidence of 4.3%. Hence the present study is statistically underpowered to confirm modest reno-protective effects. Still, both point estimates tilt toward benefit and dovetail with the 30–39% kidney-risk reductions reported in CREDENCE (canagliflozin) and DAPA-CKD (dapagliflozin) [30,31], underscoring the need for larger, longer-term Japanese cohorts.

In every analytic tier—including sensitivity and subgroup sets—the point estimate for the composite outcome favoured SGLT2 inhibitor initiation (HR = 0.80), but the confidence interval was wide (0.51–1.24) and crossed unity. Post-hoc power calculations indicate that our study, with only 79 composite events, had roughly 17% power to detect a 20% relative risk reduction; ~ 630 events would have been needed for 80% power. Consequently, the absence of statistical significance reflects insufficient sample size rather than definitive equivalence, and larger Japanese cohorts or longer follow-up will be necessary to clarify whether the apparent 20% hazard reduction is real. Until such evidence emerges, metformin remains the default first-line agent because its clinical benefits are comparable and its daily cost is markedly lower.This study has several caveats that temper the interpretation of its findings. First, as a retrospective cohort analysis, it remains vulnerable to residual bias and unmeasured confounding, limiting causal inference even after propensity-score matching. Second, key lifestyle variables—such as diet and habitual physical activity—were not available in the database; their omission could have influenced outcomes. Third, by restricting the analysis to patients who remained on their index monotherapy for 12 months and completed a health check-up within 6 months of treatment initiation, we may have introduced immortal-time bias and selected a healthier, more adherent subgroup. Both phenomena could dilute true event rates and overstate between-class cost differences, and may limit the generalizability of our findings to patients less engaged in routine health monitoring. Fourth, the exclusion criteria may have preferentially removed patients with more severe disease or complex comorbidities, introducing selection bias. Fifth, outcome ascertainment relied on ICD-10 codes, which may miss early or asymptomatic complications (e.g., microalbuminuria or subclinical neuropathy), potentially leading to underestimation of event rates and attenuating between-group differences. Sixth, longitudinal HbA1c trajectories could not be analysed because follow-up laboratory data were only captured for individuals who underwent subsequent health check-ups. Finally, all data originated from the SKDB, a single-prefecture claims resource, so the results may not generalise to other regions, healthcare systems, or ethnic groups with different genetic or physiological characteristics.

## Conclusions

Using a large-scale regional database from Japan reflecting routine clinical practice, we found that, among adults with type 2 diabetes without prior major cardiac or renal disease who began monotherapy with either a biguanide or an SGLT2 inhibitor and avoided the alternate class during the first year (while allowing any other concomitant drugs), first‑line SGLT2 treatment did not lower risks of cardio‑cerebrovascular events, all‑cause mortality, or diabetic complications compared with metformin and incurred approximately 50% higher costs.

## Supporting information

S1 Table Search codes for SGLT2 inhibitors and biguanides. SGLT2: Sodium glucose cotransporter 2.
(DOCX)

S2 Table Definitions of comorbidities using ICD-10 codes. ICD-10: International Classification of Diseases, 10th Revision.
(DOCX)

S3 Table ATC codes for other antidiabetic medication. ATC: anatomical therapeutic chemical, DPP-4: dipeptidyl peptidase-4, GLP-1: glucagon-like peptide-1, SGLT2: Sodium–glucose cotransporter 2.
(DOCX)

S4 Table Definitions of outcomes. ICD-10: International Classification of Diseases, 10th Revision.
(DOCX)

S5 Table Diagnostic codes for diabetes in Japan. ICD-10: International Classification of Diseases, 10th Revision.
(DOCX)

S6 Table Characteristics of the participants prior to propensity score matching. ALT, alanine aminotransferase; AST, aspartate aminotransferase; BMI, body mass index; GFR, glomerular filtration rate; GGT, gamma-glutamyl transpeptidase; HbA1c, hemoglobin A1c; LDL, low-density lipoprotein; HDL, high-density lipoprotein; SGLT2, Sodium glucose cotransporter 2; ASMD: absolute standardized mean difference.
(DOCX)

S7 Table Antidiabetic medication prescribed and the number of health care visits within the year following the index date, before propensity score matching. DPP-4: dipeptidyl peptidase-4; GLP-1: glucagon-like peptide-1; SGLT2: sodium–glucose cotransporter 2.
(DOCX)

S8 Table Outcomes of the participants who were prescribed biguanide or a SGLT2 inhibitor in the matched cohort and who attended the clinic for ≥9 months, as a sensitivity analysis (n = 1,046). *Gray’s test was performed. †The log-rank test was performed. SGLT2: Sodium glucose cotransporter 2 inhibitor.
(DOCX)

S9 Table Outcomes of participants who were prescribed biguanide or a SGLT2 inhibitor in the matched cohort and who had attended the clinic for ≥12 months, as a sensitivity analysis (n = 582). *Gray’s test was performed. †The log-rank test was performed. SGLT2: Sodium glucose cotransporter 2 inhibitor.
(DOCX)
