# Supplementary material for: Comparative effectiveness of biguanides versus SGLT2 inhibitors on cardiovascular and cerebrovascular events, diabetic nephropathy, retinopathy, neuropathy, and treatment expenditures in patients with type 2 diabetes
Source: PLoS One. 2025 Nov 6;20(11):e0336038. doi: 10.1371/journal.pone.0336038 (PMC12591428; doi:10.1371/journal.pone.0336038)
Supplement: S1 Table — SGLT2: Sodium glucose cotransporter 2. (DOCX) [file pone.0336038.s001.docx]

**S1 Table.** Search codes for SGLT2 inhibitors and biguanides.

| **Medication** | **Code** |
| --- | --- |
| **SGLT2 inhibitor/combination** |  |
| Ipragliflozin L-proline | 622306601, 622306701 |
| Tofogliflozin Hydrate | 622335701, 622335801 |
| Luseogliflozin Hydrate | 622336801, 622340101, 622341901, 622342001 |
| Canagliflozin Hydrate | 622360601 |
| Empagliflozin | 622401201, 622401301 |
| Canagliflozin Hydrate /  Teneligliptin Hydrobromide Hydrate (DPP-4i) | 622573601 |
| Ipragliflozin L-proline /  Sitagliptin Phosphate Hydrate (DPP-4i) | 622625702 |
| Empagliflozin / Linagliptin (DPP-4i) | 622655001, 622655101 |
| **Biguanides** |  |
| Buformin hydrochloride | 620004502, 620005979, 620873901 |
| Metformin hydrochloride | 610444147, 610463145, 620002859, 620004480, 620005570, 621676001, 621974701, 622070801, 622242501, 622412701, 622417101, 622417201, 622421101, 622421201, 622421901, 622422001, 622424401, 622424501, 622427201, 622427301, 622432601, 622432701, 622436301, 622438401, 622438501, 622448601, 622466601, 622784601, 622784701, 622822401, 622822501 |

SGLT2: Sodium glucose cotransporter 2.
