# Supplementary material for: Comparative effectiveness of biguanides versus SGLT2 inhibitors on cardiovascular and cerebrovascular events, diabetic nephropathy, retinopathy, neuropathy, and treatment expenditures in patients with type 2 diabetes
Source: PLoS One. 2025 Nov 6;20(11):e0336038. doi: 10.1371/journal.pone.0336038 (PMC12591428; doi:10.1371/journal.pone.0336038)
Supplement: S2 Table — ICD-10: International Classification of Diseases, 10th Revision. (DOCX) [file pone.0336038.s002.docx]

**S2 Table.** Definitions of comorbidities using ICD-10 codes

| **Comorbidity** | **ICD-10 codes** |
| --- | --- |
| Hypertension | I10, I11, I12, I13, I15 |
| Dementia | F00, F01, F02, F03, F051, G30, G311 |
| Renal disease | I120, I131, N032, N033, N034, N035, N036, N037, N052, N053, N054, N055, N056, N057, N18, N19, N250, Z490, Z491, Z492, Z940, Z992 |
| Rheumatic disease | L940, L941, L943, M05, M06, M08, M120, M123, M30, M310, M311, M312, M313, M315, M32, M33, M34, M35, M360, M45, M461, M468, M469 |
| Liver disease | B18, I85, I864, I982, K70, K711, K713, K714, K715, K717, K72, K73, K74, K760, K762, K763, K764, K765, K766, K767, K768, K769, Z944 |
| Chronic pulmonary disease | I278, I279, J40, J41, J42, J43, J44, J45, J46, J47, J60, J61, J62, J63, J64, J65, J66, J67, J684, J701, J703 |
| Diabetes | E100, E101, E102, E103, E104, E105, E106, E107, E108, E109, E110, E111, E112, E113, E114, E115, E116, E117, E118, E119, E120, E121, E122, E123, E124, E125, E126, E127, E128, E129, E130, E131, E132, E133, E134, E135, E136, E137, E138, E139, E140, E141, E142, E143, E144, E145, E146, E147, E148, E149 |

ICD-10: International Classification of Diseases, 10th Revision.
