# Supplementary material for: Comparative effectiveness of biguanides versus SGLT2 inhibitors on cardiovascular and cerebrovascular events, diabetic nephropathy, retinopathy, neuropathy, and treatment expenditures in patients with type 2 diabetes
Source: PLoS One. 2025 Nov 6;20(11):e0336038. doi: 10.1371/journal.pone.0336038 (PMC12591428; doi:10.1371/journal.pone.0336038)
Supplement: S4 Table — ICD-10: International Classification of Diseases, 10th Revision. (DOCX) [file pone.0336038.s004.docx]

**S4 Table.** Definitions of outcomes.

| **Outcome** | **ICD-10 or disease code** | **Detail** |
| --- | --- | --- |
| Cardiac event | I20, I21, I22, I24 as acute coronary syndromes  I25 as chronic heart disease  I50 as heart failure | Of these coded participants, only those who were hospitalized were considered to have experienced events |
|  | 150260350, 150284310, 150318310, 150359310, 150153910, 150374910, 150375010, 150375110, 150263310, 150375210, 150375310, 150375410, 150145910, 150146010, 150318410, 150318510 | Percutaneous coronary intervention or coronary artery bypass grafting |
| Cerebrovascular event | I60, I61, I62, I63, I64 as stroke  8838736, 8838748, 8838750 as another cerebrovascular event | Of these coded patients, only those who were hospitalized were considered to have experienced events |

ICD-10: International Classification of Diseases, 10th Revision.
