# Supplementary material for: Comparative effectiveness of biguanides versus SGLT2 inhibitors on cardiovascular and cerebrovascular events, diabetic nephropathy, retinopathy, neuropathy, and treatment expenditures in patients with type 2 diabetes
Source: PLoS One. 2025 Nov 6;20(11):e0336038. doi: 10.1371/journal.pone.0336038 (PMC12591428; doi:10.1371/journal.pone.0336038)
Supplement: S5 Table — ICD-10: International Classification of Diseases, 10th Revision. (DOCX) [file pone.0336038.s005.docx]

**S5 Table.** Diagnostic codes for diabetic complications in Japan.

| **Outcome** | **Disease code** | **ICD-10** | **Disease name** |
| --- | --- | --- | --- |
| Diabetic retinopathy | 8830045 | E113 | Type 2 diabetic retinopathy |
|  | 8844537 | E113 | Proliferative diabetic retinopathy, type 2 diabetes |
|  | 8845093 | E113 | Type 2 diabetic central retinopathy |
|  | 2504005 | E143 | Diabetic central retinopathy |
|  | 2504010 | E143 | Proliferative diabetic retinopathy |
|  | 2504013 | E143 | Diabetic retinopathy |
| Diabetic nephropathy | 8830042 | E112 | Type 2 diabetic nephropathy |
|  | 8841691 | E112 | Type 2 diabetes mellitus with diabetic nephropathy |
|  | 8843993 | E112 | Type 2 diabetic nephropathy phase 3 |
|  | 8843994 | E112 | Type 2 diabetic nephropathy phase 3A |
|  | 8843995 | E112 | Type 2 diabetic nephropathy phase 3B |
|  | 8843996 | E112 | Type 2 diabetic nephropathy phase 4 |
|  | 8843997 | E112 | Type 2 diabetic nephropathy phase 5 |
|  | 8845087 | E112 | Type 2 diabetic nephrosclerosis |
|  | 8845088 | E112 | Type 2 diabetic kidney failure |
|  | 2503005 | E142 | Diabetic nephropathy |
|  | 2503007 | E142 | Diabetic renal failure |
|  | 8832747 | E142 | Kimmelstiel–Wilson syndrome |
|  | 8838071 | E142 | Diabetic nephrosclerosis |
|  | 8850065 | E142 | Diabetic kidney disease |
| Diabetic neuropathy | 8830043 | E114 | Type 2 diabetic neuropathy |
|  | 8830044 | E114 | Type 2 diabetic myopathy |
|  | 8841693 | E114 | Type 2 diabetes mellitus with neurological complications |
|  | 8845079 | E114 | Type 2 diabetic muscular atrophy |
|  | 8845084 | E114 | Type 2 diabetic neuropathic bladder |
|  | 8845085 | E114 | Type 2 diabetic neuralgia |
|  | 8845086 | E114 | Type 2 diabetic autonomic neuropathy |
|  | 8845091 | E114 | Type 2 diabetic polyneuropathy |
|  | 8845092 | E114 | Type 2 diabetic mononeuropathy |
|  | 8845100 | E114 | Type 2 diabetic peripheral neuropathy |
|  | 2505011 | E144 | Diabetic neuralgia |
|  | 2505018 | E144 | Diabetic peripheral neuropathy |
|  | 2505021 | E144 | Diabetic muscular atrophy |
|  | 8838069 | E144 | Diabetic neuropathic bladder |
|  | 8838070 | E144 | Diabetic autonomic neuropathy |
|  | 8838074 | E144 | Diabetic polyneuropathy |
|  | 8838075 | E144 | Diabetic mononeuropathy |
|  | 8838078 | E144 | Diabetic neuropathy |
|  | 8848768 | E144 | Diabetic neuropathic pain |
| Other conditions | 8830041 | E110 | Type 2 diabetic coma |
|  | 8845094 | E110 | Hypoglycemic coma in the context of type 2 diabetes mellitus |
|  | 8841692 | E113 | Type 2 diabetes mellitus with ocular complications |
|  | 8843990 | E113 | Type 2 diabetic macular edema |
|  | 8844347 | E113 | Type 2 diabetic cataract |
|  | 8845072 | E113 | Type 2 diabetic macular edema |
|  | 8845078 | E113 | Type 2 diabetic ocular muscle paralysis |
|  | 8845082 | E113 | Type 2 diabetic iritis |
|  | 8841694 | E115 | Type 2 diabetes mellitus with peripheral circulation complications |
|  | 8843106 | E115 | Type 2 diabetic gangrene |
|  | 8845075 | E115 | Type 2 diabetic ulcer |
|  | 8845080 | E115 | Type 2 diabetic vascular disease |
|  | 8845095 | E115 | Type 2 diabetic atherosclerosis |
|  | 8845096 | E115 | Type 2 diabetic arterial occlusion |
|  | 8845098 | E115 | Type 2 diabetic peripheral vascular disease |
|  | 8845099 | E115 | Type 2 diabetic peripheral vascular disease |
|  | 8841695 | E116 | Type 2 diabetes mellitus with joint complications |
|  | 8841696 | E116 | Type 2 diabetes mellitus with diabetic complications |
|  | 8844628 | E116 | Type 2 diabetic blister |
|  | 8844629 | E116 | Type 2 diabetic edematous sclerosis |
|  | 8845076 | E116 | Type 2 diabetic liver injury |
|  | 8845077 | E116 | Type 2 diabetic arthropathy |
|  | 8845081 | E116 | Type 2 diabetic hypercholesterolemia |
|  | 8845083 | E116 | Type 2 diabetic osteopathy |
|  | 8845089 | E116 | Type 2 diabetic mental disorder |
|  | 8845090 | E116 | Type 2 diabetic pruritus |
|  | 8845097 | E116 | Type 2 diabetic skin disorder |
|  | 8848108 | E116 | Type 2 diabetic gastroenteritis |
|  | 8849558 | E116 | Type 2 diabetic hyperosmolar hyperglycemic syndrome |
|  | 8841697 | E117 | Type 2 diabetes mellitus with multiple diabetic complications |
|  | 2502004 | E140 | Diabetic nonketotic hyperosmolar coma |
|  | 2502006 | E140 | Diabetic coma |
|  | 2504004 | E143 | Diabetic iritis |
|  | 2504006 | E143 | Diabetic cataract |
|  | 2504012 | E143 | Diabetic maculopathy |
|  | 8838065 | E143 | Diabetic ophthalmoplegia |
|  | 8844089 | E143 | Diabetic macular edema |
|  | 8848634 | E144 | Diabetic foot lesion |
|  | 2506006 | E145 | Diabetic gangrene |
|  | 2506011 | E145 | Diabetic arterial occlusion |
|  | 8838063 | E145 | Diabetic ulcer |
|  | 8838066 | E145 | Diabetic angiopathy |
|  | 8838077 | E145 | Diabetic arteriosclerosis |
|  | 8838079 | E145 | Diabetic peripheral vascular disease |
|  | 8838080 | E145 | Diabetic peripheral vascular disease |
|  | 8848632 | E145 | Diabetic foot gangrene |
|  | 8848633 | E145 | Diabetic foot ulcer |
|  | 2507025 | E146 | Diabetic arthropathy |
|  | 2507029 | E146 | Diabetic skin disorders |
|  | 8838064 | E146 | Diabetic liver injury |
|  | 8838067 | E146 | Diabetic hypercholesterolemia |
|  | 8838068 | E146 | Diabetic osteopathy |
|  | 8838072 | E146 | Diabetic mental disorder |
|  | 8838073 | E146 | Diabetic pruritus |
|  | 8844652 | E146 | Diabetic blister |
|  | 8844653 | E146 | Diabetic edematous sclerosis |

ICD-10: International Classification of Diseases, 10th Revision.
