# Supplementary material for: Comparative effectiveness of biguanides versus SGLT2 inhibitors on cardiovascular and cerebrovascular events, diabetic nephropathy, retinopathy, neuropathy, and treatment expenditures in patients with type 2 diabetes
Source: PLoS One. 2025 Nov 6;20(11):e0336038. doi: 10.1371/journal.pone.0336038 (PMC12591428; doi:10.1371/journal.pone.0336038)
Supplement: S7 Table — DPP-4: dipeptidyl peptidase-4; GLP-1: glucagon-like peptide-1; SGLT2: sodium–glucose cotransporter 2. (DOCX) [file pone.0336038.s007.docx]

**S7 Table.** Antidiabetic medication prescribed and the number of health care visits within the year following the index date, before propensity score matching.

| **Variable** | **Before matching** | |
| --- | --- | --- |
|  | **Biguanide**  **(N=1,586)** | **SGLT2 inhibitor**  **(n=1,050)** |
| Biguanides | 1050 (100.0) | 0 |
| SGLT2 inhibitors | 0 | 1586 (100.0) |
| Insulin | 0 | 0 |
| GLP-1 receptor agonists | 1 (0.1) | 7 (0.7) |
| DPP-4 inhibitors | 1071 (67.5) | 601 (57.2) |
| Alpha-glucosidase inhibitors | 145 (9.1) | 87 (8.3) |
| Thiazolidinediones (also known as glitazones) | 86 (5.4) | 78 (7.4) |
| Rapid-acting secretagogues  (meglitinides, also known as glinides) | 69 (4.4) | 54 (5.1) |
| Sulfonylureas | 261 (16.5) | 222 (21.1) |
| Number of healthcare visits per year | 10.66 (1.81) | 10.58 (1.84) |

DPP-4: dipeptidyl peptidase-4, GLP-1: glucagon-like peptide-1; SGLT2: sodium-glucose cotransporter 2
