# Supplementary material for: Comparative effectiveness of biguanides versus SGLT2 inhibitors on cardiovascular and cerebrovascular events, diabetic nephropathy, retinopathy, neuropathy, and treatment expenditures in patients with type 2 diabetes
Source: PLoS One. 2025 Nov 6;20(11):e0336038. doi: 10.1371/journal.pone.0336038 (PMC12591428; doi:10.1371/journal.pone.0336038)
Supplement: S8 Table — *Gray’s test was performed. †The log-rank test was performed. SGLT2: Sodium glucose cotransporter 2 inhibitor. (DOCX) [file pone.0336038.s008.docx]

**S8** **Table.** Outcomes of the participants who were prescribed biguanide or a SGLT2 inhibitor in the matched cohort and who attended the clinic for ≥9 months, as a sensitivity analysis (n=1,046).

| **Outcome** | **Exposure** | **Events, number (%)** | **Cumulative incidence after 3 years** | | ***P*-value** |
| --- | --- | --- | --- | --- | --- |
|  |  |  | **Rate** | **95% Confidence interval** |  |
| Composite event**^†^** | Biguanide (n = 523) | 48 (9.2) | 6.6 | 4.6 - 9.6 | 0.115 |
|  | SGLT2 inhibitor (n = 523) | 33 (6.3) | 6.4 | 4.3 - 9.3 |  |
| Cardiac event^*^ | Biguanide (n = 523) | 31 (5.9) | 4.3 | 2.6 - 6.7 | **0.033** |
|  | SGLT2 inhibitor (n = 523) | 16 (3.1) | 3.0 | 1.6 - 5.0 |  |
| Cerebrovascular event^*^ | Biguanide (n = 523) | 15 (2.9) | 2.2 | 1.1 - 4.0 | 0.483 |
|  | SGLT2 inhibitor (n = 523) | 11 (2.1) | 2.4 | 1.2 - 4.3 |  |
| Death**^†^** | Biguanide (n = 523) | 11 (2.1) | 1.2 | 0.5 - 2.8 | 0.697 |
|  | SGLT2 inhibitor (n = 523) | 12 (2.3) | 2.0 | 1.0 - 4.1 |  |
| Diabetic complication^*^ | Biguanide (n = 523) | 77 (14.7) | 15.0 | 11.5 - 18.8 | 0.405 |
|  | SGLT2 inhibitor (n = 523) | 70 (13.4) | 13.2 | 10.0 - 16.9 |  |
| Diabetic retinopathy^*^ | Biguanide (n = 523) | 57 (10.9) | 11.4 | 8.4 – 14.9 | 0.942 |
|  | SGLT2 inhibitor (n = 523) | 58 (11.1) | 10.6 | 7.7 - 13.9 |  |
| Diabetic nephropathy^*^ | Biguanide (n = 523) | 21 (4.0) | 3.1 | 1.7 – 5.0 | 0.254 |
|  | SGLT2 inhibitor (n = 523) | 14 (2.7) | 2.4 | 1.2 – 4.3 |  |
| Diabetic neuropathy^*^ | Biguanide (n = 523) | 8 (1.5) | 1.0 | 0.3- 2.3 | 0.477 |
|  | SGLT2 inhibitor (n = 523) | 5 (1.0) | 0.4 | 0.1 – 1.4 |  |
| Other conditions^*^ | Biguanide (n = 523) | 10 (1.9) | 2.4 | 1.2 - 4.3 | 0.656 |
|  | SGLT2 inhibitor (n = 523) | 8 (1.5) | 1.1 | 0.4- 2.6 |  |

^*^Gray’s test was performed. ^†^The log-rank test was performed. SGLT2: Sodium glucose cotransporter 2 inhibitor.
