# Supplementary material for: Comparative effectiveness of biguanides versus SGLT2 inhibitors on cardiovascular and cerebrovascular events, diabetic nephropathy, retinopathy, neuropathy, and treatment expenditures in patients with type 2 diabetes
Source: PLoS One. 2025 Nov 6;20(11):e0336038. doi: 10.1371/journal.pone.0336038 (PMC12591428; doi:10.1371/journal.pone.0336038)
Supplement: S9 Table — *Gray’s test was performed. †The log-rank test was performed. SGLT2: Sodium glucose cotransporter 2 inhibitor. (DOCX) [file pone.0336038.s009.docx]

**S9** **Table.** Outcomes of participants who were prescribed biguanide or a SGLT2 inhibitor in the matched cohort and who had attended the clinic for ≥12 months, as a sensitivity analysis (n=582).

| **Outcome** | **Exposure** | **Events (%)** | **Cumulative incidence after 3 years** | | ***P*-value** |
| --- | --- | --- | --- | --- | --- |
|  |  |  | **Rate** | **95% Confidence interval** |  |
| Composite event**^†^** | Biguanide (n = 291) | 33 (11.3) | 6.0 | 3.6 - 9.8 | 0.148 |
|  | SGLT2 inhibitor (n = 291) | 17 (5.8) | 6.4 | 3.9 – 10.6 |  |
| Cardiac event^*^ | Biguanide (n = 291) | 23 (7.8) | 4.1 | 2.1 - 7.2 | 0.325 |
|  | SGLT2 inhibitor (n = 291) | 10 (3.4) | 3.8 | 1.9 – 6.9 |  |
| Cerebrovascular event^*^ | Biguanide (n = 291) | 9 (3.1) | 1.4 | 0.5 - 3.4 | 0.484 |
|  | SGLT2 inhibitor (n = 291) | 6 (2.0) | 2.2 | 0.8 – 4.9 |  |
| Death**^†^** | Biguanide (n = 291) | 7 (2.4) | 1.2 | 0.4 – 3.8 | 0.160 |
|  | SGLT2 inhibitor (n = 291) | 3 (1.0) | 0.5 | 0.1 - 3.3 |  |
| Diabetic complication^*^ | Biguanide (n = 291) | 45 (15.4) | 16.7 | 11.9 - 22.2 | 0.753 |
|  | SGLT2 inhibitor (n = 291) | 39 (13.3) | 13.9 | 9.7 – 18.8 |  |
| Diabetic retinopathy^*^ | Biguanide (n = 291) | 28 (9.6) | 10.5 | 6.8 – 15.2 | 0.151 |
|  | SGLT2 inhibitor (n = 291) | 31 (10.6) | 11.3 | 7.5 - 15.8 |  |
| Diabetic nephropathy^*^ | Biguanide (n = 291) | 17 (5.8) | 4.3 | 2.3 – 7.4 | **0.034** |
|  | SGLT2 inhibitor (n = 291) | 8 (2.7) | 2.5 | 1.0 - 5.2 |  |
| Diabetic neuropathy^*^ | Biguanide (n = 291) | 7 (2.4) | 1.6 | 0.5 – 3.8 | 0.173 |
|  | SGLT2 inhibitor (n = 291) | 2 (0.7) | 0.4 | <0.01 - 1.9 |  |
| Other conditions^*^ | Biguanide (n = 291) | 9 (3.1) | 3.4 | 1.5 – 6.5 | 0.579 |
|  | SGLT2 inhibitor (n = 291) | 4 (1.4) | 1.2 | 0.3 – 3.2 |  |

^*^Gray’s test was performed. ^†^The log-rank test was performed. SGLT2: Sodium glucose cotransporter 2 inhibitor.
